# Iron Overload in Hematopoietic Stem Cell Transplantation

**DOI:** 10.4274/tjh.2016.0493

**Published:** 2017-03-01

**Authors:** Sora Yasri, Viroj Wiwanitkit

**Affiliations:** 1 KMT Primary Care Center, Bangkok, Thailand; 2 Wiwanitkit House, Bangkok, Thailand

**Keywords:** iron, Overload, Hematopoietic stem cell, Transplantation

## TO THE EDITOR,

We read the publication entitled “Current Review of Iron Overload and Related Complications in Hematopoietic Stem Cell Transplantation” with great interest [1]. As summarized by Atilla et al. [1], “Organ dysfunction due to iron overload may cause high mortality rates and therefore a sufficient iron chelation therapy is recommended”. We would like to share the experience from our settings where there is a very high prevalence of thalassemia and transplantation is the only curative treatment.

Iron overload is common among transfusion-dependent thalassemia patients and transfusion during transplantation might increase the risk of the complication of iron overload. However, in clinical practice, the problem is not common and improvement of the patients after transplantation is reported. According to the recent report by Inati et al. [2], with standard chelation therapy, the outcome of thalassemic patients undergoing stem cell transplantation is usually favorable. The use of the standard dosage of deferoxamine, with or without phlebotomy, accompanied with close iron status monitoring can be effective [2,3]. It can be seen that stem cell transplantation can be problematic despite there being a need of hypertransfusion during the process even though the patient might have an underlying severe iron overload condition such as thalassemia.
