# Supplementary material for: Iron Overload in Hematopoietic Stem Cell Transplantation
Source: Turk J Haematol. 2017 Mar 1;34(1):102–3. doi: 10.4274/tjh.2016.0493 (PMC5451671; doi:10.4274/tjh.2016.0493)
Supplement: Related texts

# Reply: Authors' Response

**Authors:** Erden Atilla, Selami K. Toprak, Taner Demirer

Dear Sora Yasri,

Thank you very much for your valuable comments and sharing your experience. We agree for your contribution. In thalassemia patients, several transplantation centers categorised risk factors prior to allogenic hematopoietic stem cell transplantation. Pesaro classification assigned patients to three arms according to the absence or presence of one, two or three risk factors: hepatomegaly > 2 cm, portal fibrosis, and irregular chelation history [1]. It should be kept in mind that in a study by Ghavamzadeh et al., liver iron overload did not change after transplant (p=0.61) but hepatic fibrosis progressed (p=0.01) [2]. Allogeneic stem cell transplantation did not reduce liver iron overload and in fact liver fibrosis increased. Also steps for reducing iron overload should be taken in the post transplant setting [3]. Iron overload is still an essential issue in both pre and post transplant settings. Survival in transfusion-dependent thalassemia patients can be improved with proper understanding of the pathophysiology of thalassemia and iron toxicity.

Regards,

## References

[1] Karas-Kuzelicki N, Lucarelli G, Weatherall DJ (1991). FFor debate: bone marrow transplantation for severe thalassaemia (1). The view from Pesaro (2). To be or not to be. Br J Haematol.

[2] Ghavamzadeh A, Mirzania M, Kamalian N, Sedighi N, Azimi P (2015). Hepatic iron overload and fibrosis in patients with beta thalassemia major after hematopoietic stem cell transplantation: a pilot study. Int J Hematol Oncol Stem Cell Res.

[3] Bayanzay K, Alzoebie L (2016). Reducing the iron burden and improving survival in transfusion-dependent thalassemia patients: current perspectives. J Blood Med.
